# Comprehensive Analysis of Cell Cycle-Related Genes in Patients With Prostate Cancer

**DOI:** 10.3389/fonc.2021.796795

**Published:** 2022-01-11

**Authors:** Zehua Liu, Rongfang Pan, Wenxian Li, Yanjiang Li

**Affiliations:** ^1^ Department of Urology, The Affiliated Hospital of Qingdao University, Qingdao, China; ^2^ Department of Nutrition, The Affiliated Hospital of Qingdao University, Qingdao, China

**Keywords:** prostate cancer, GSVA, cell cycle, recurrence-free survival, immunotherapy

## Abstract

This study aimed to identify critical cell cycle-related genes (CCRGs) in prostate cancer (PRAD) and to evaluate the clinical prognostic value of the gene panel selected. Gene set variation analysis (GSVA) of dysregulated genes between PRAD and normal tissues demonstrated that the cell cycle-related pathways played vital roles in PRAD. Patients were classified into four clusters, which were associated with recurrence-free survival (RFS). Moreover, 200 prognostic-related genes were selected using the Kaplan–Meier (KM) survival analysis and univariable Cox regression. The prognostic CCRG risk score was constructed using random forest survival and multivariate regression Cox methods, and their efficiency was validated in Memorial Sloan Kettering Cancer Center (MSKCC) and GSE70770. We identified nine survival-related genes: CCNL2, CDCA5, KAT2A, CHTF18, SPC24, EME2, CDK5RAP3, CDC20, and PTTG1. Based on the median risk score, the patients were divided into two groups. Then the functional enrichment analyses, mutational profiles, immune components, estimated half-maximal inhibitory concentration (IC50), and candidate drugs were screened of these two groups. In addition, the characteristics of nine hub CCRGs were explored in Oncomine, cBioPortal, and the Human Protein Atlas (HPA) datasets. Finally, the expression profiles of these hub CCRGs were validated in RWPE-1 and three PRAD cell lines (PC-3, C4-2, and DU-145). In conclusion, our study systematically explored the role of CCRGs in PRAD and constructed a risk model that can predict the clinical prognosis and immunotherapeutic benefits.

## Introduction

Prostate cancer (PRAD) is the most common carcinoma in men (1) and a significant global health concern (2). The main treatment for PRAD is radical prostatectomy (3). Although most PRAD patients could benefit from this treatment, nearly 27%–53% of patients who have undergone this procedure will progress into advanced PRAD and castration-resistant prostate cancer (CRPC) (4, 5). Therefore, timely diagnosis of PRAD and exploring the detailed mechanisms involved in PRAD are critical for improving the prognosis of patients with PRAD.

The cell cycle is one of the vital biological processes in the organism (6). The cell cycle regulates the process of cell division and duplication of genetic materials (7), which is highly associated with the growth and proliferation of cancer cells. Increasing numbers of studies showed that certain genes and drugs serve as potential cycle regulators. For instance, in prostate cancer cells, upregulation of PHLDA3 inhibited cell proliferation by inducing cell cycle arrest at G1 *via* a decrease in AKT phosphorylation and activation of Wnt/β-catenin (8). The decreased expression of SMARCC1 dramatically accelerated prostate cancer cell proliferation by enhancing cell cycle progression (9). Platycodin D could promote sorafenib-induced apoptosis and cell cycle arrest in prostate cancer cells (10) and BTT-3033 attenuated prostate cancer cell viability and proliferation by cell cycle arrest (11). Thus, cell cycle arrest may be used as a novel therapeutic strategy. However, such research is a lengthy process whose results do not immediately translate into clinical practice.

In this study, we firstly identified the molecular hallmarks in normal samples and PRAD tissues using the Gene Set Enrichment Analysis (GSEA) method. The results showed that cell cycle-related pathways, such as DNA_REPAIR, E2F_TARGETs, G2M_CHECKPOINT, MYC _TARGETS_V1, and MYC_TARGETS_V2, were enriched in the PRAD tissues. Considering the critical roles of cell cycle-related genes (CCRGs) in the initiation and progression of cancers, then we hypothesized that CCRGs may provide a novel insight into the treatment and prognosis of PRAD. Therefore, there is an urgent need to explore the roles of CCRGs in the prognosis and treatment of PRAD patients.

## Results

### Functional Pathway Screening Using Gene Set Variation Analysis

**Figure 1:**
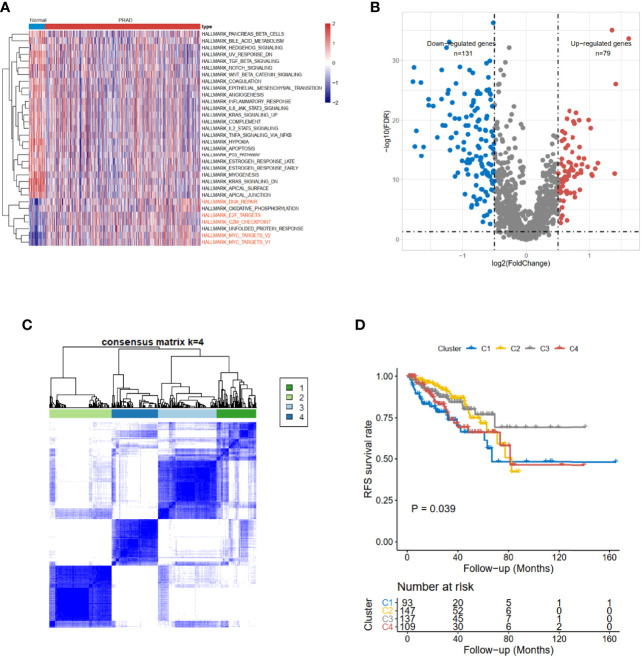
Identification of cell cycle-related DEGs and consensus clustering in TCGA-PRAD cohort. **(A)** The hallmark enrichment of prostate cancer compared with normal tissues. **(B)** The volcano plot of cell cycle-related DEGs between high- and low-risk groups (FDR < 0.05 and logFC > 0.5). **(C)** Consensus matrices for a solution with 4 clusters based on DEGs. **(D)** Kaplan–Meier curves show the prognostic value of the four patterns. DEGs, differentially expressed genes; TCGA-PRAD, The Cancer Genome Atlas—Prostate Cancer; FDR, false discovery rate.

The clinical information of 551 subjects, including 499 PRAD patients and 52 healthy volunteers, was downloaded from UCSC Xena. Based on The Cancer Genome Atlas (TCGA)-PRAD cohort data, gene set variation analysis (GSVA) results showed the CCRG sets, such as HALLMARK_DNA_REPAIR, HALLMARK_E2F_TARGETs, HALLMARK_G2M_CHECKPOINT, and HALLMARK_MYC_TARGETS_V1 (**Figure 1A**) were enriched in PRAD patients.

### Cluster Analysis Based on Cell Cycle-Related Genes

The limma (12) R package was used to detect differentially expressed CCRGs. The volcano map of the differentially expressed CCRGs showed that there were 79 upregulated genes and 131 downregulated genes (**Figure 1B**). TCGA-PRAD cohort could be divided into four clusters based on differentially expressed CCRGs (**Figure 1C**). Moreover, patients had significant differences among these four clusters (*p* < 0.039, **Figure 1D**).

### Construction and Validation of Cell Cycle-Related Gene Prognostic Model

**Figure 2:**
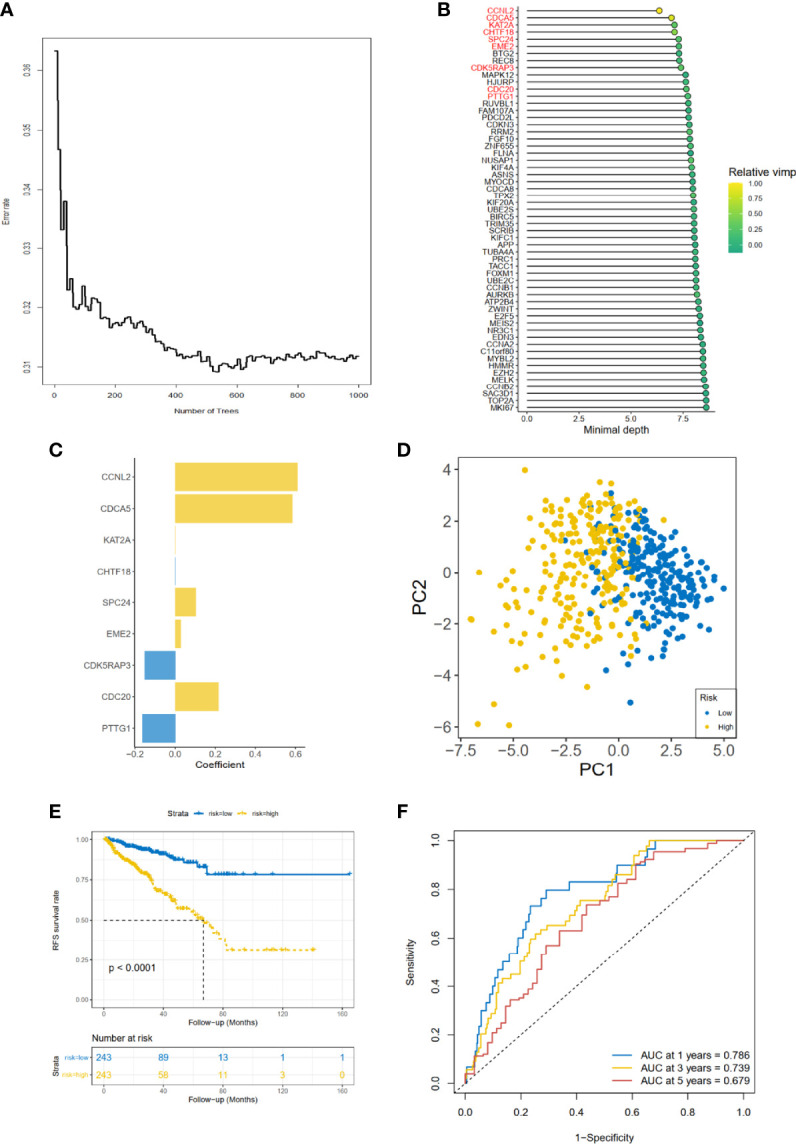
Gene selection and risk prognostic model of CCRGs based on TCGA cohort. **(A)** Estimation of the OOB prediction error rate based on the random forest. **(B)** The top 15 genes according to both minimal depth and variable importance. **(C)** The coefficient for genes of risk prognostic model. **(D)** The distribution of risk scores based on PCA. The high-risk group was annotated by yellow and the low-risk group by blue. **(E)** Kaplan–Meier curves of RFS stratified by risk score. **(F)** Time-dependent ROC curve analysis for risk score. CCRGs, cell cycle-related genes; TCGA, The Cancer Genome Atlas; OOB, out of bag; PCA, principal component analysis; RFS, recurrence-free survival; ROC, receiver operating characteristic.

**Figure 3:**
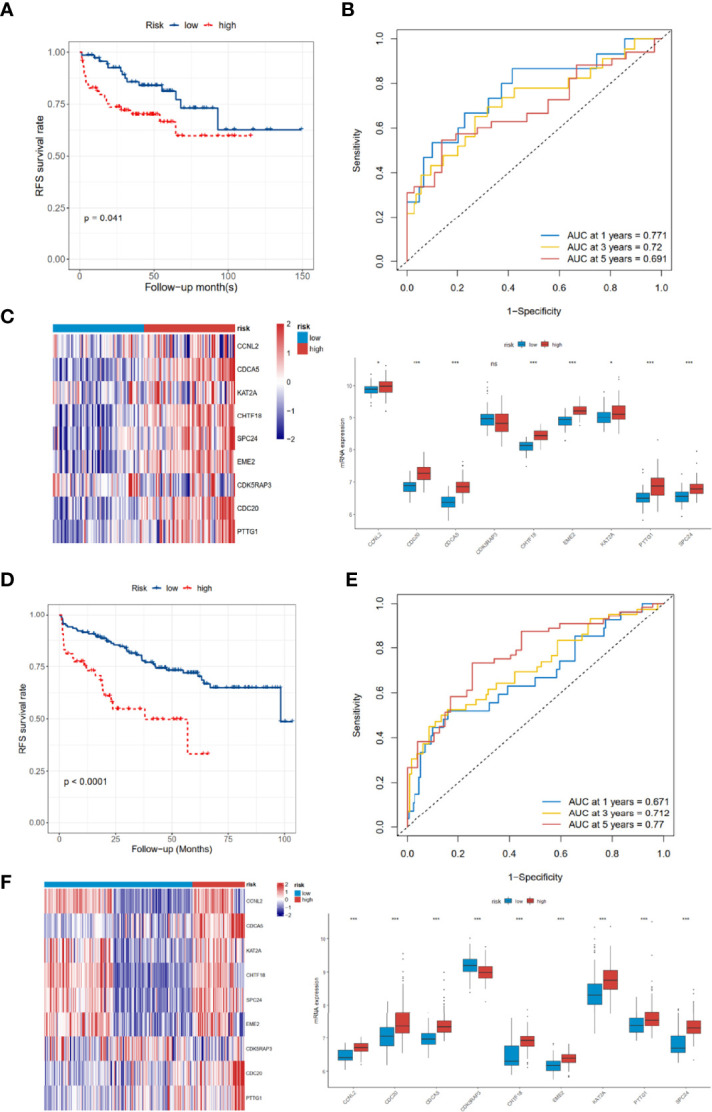
Validation of the 9-gene signature in the MSKCC and GSE70770 cohort. **(A–C)** The distribution of RFS, RFS status, KM curves, and ROC curves in the MSKCC cohort. **(D–F)** The distribution of RFS, RFS status, KM curves, and ROC curves in the GSE70770 cohort. MSKCC, Memorial Sloan Kettering Cancer Center; RFS, recurrence-free survival; KM, Kaplan–Meier; ROC, receiver operating characteristic. ns, no significance. *p < 0.05, ***p < 0.001.

As for the 210 differentially expressed CCRGs, univariate Cox regression analysis showed that 57 CCRGs were significantly associated with recurrence-free survival (RFS) in PRAD (**Table 1**). Thus, these 57 CCRGs served as input to construct a random survival forest survival model. The out-of-bag (OOB) prediction error estimator indicated that the forest prediction error tended to be steady when the number of trees was nearly 400 (**Figure 2A**). During the hub gene selection process, the top 15 ranked genes in both minimal depth and VIMP were chosen for further model construction (**Figure 2B**). Finally, CCNL2, CDCA5, KAT2A, CHTF18, SPC24, EME2, CDK5RAP3, CDC20, and PTTG1 were included in the prognosis model construction, and the risk score of each patient was calculated based on the following formula with coefficients showed in **Figure 2C**: Risk score = (0.6097 * ExpCCNL2) + (0.5850 * ExpCDCA5) + (0.0006 * ExpKAT2A) + (−0.0005 * ExpCHTF18) + (0.1037 * ExpSPC24) + (0.0283 * ExpEME2) + (−0.1527 * ExpCDK5RAP3) + (0.2168 * ExpCDC20) + (−0.1638 * ExpPTTG1). Based on the median value of the risk score, TCGA-PRAD patients were divided into high and low risk group. Principal component analysis (PCA) indicated that the two risk groups were distributed in two directions (**Figure 2D**). The Kaplan–Meier (KM) curve showed that the high-risk group patients had poorer RFS than the low-risk group (**Figure 2E**, *p* < 0.001). The prognosis performance of the risk score for RFS was assessed by time-dependent receiver operating characteristic (ROC) curves, with the area under the curve (AUC) for 1, 3, and 5 years being 0.786, 0.739, and 0.679, respectively (**Figure 2F**). Moreover, the results of two independent cohorts showed that the high-risk group was associated with worse RFS (Memorial Sloan Kettering Cancer Center (MSKCC), *p* = 0.041, and GSE70770, *p* < 0.001) (**Figures 3A, D**), which were consistent with the results in TCGA-PRAD cohort. The AUCs in MSKCC were 0.771, 0.72, and 0.691 for 1, 3, and 5 years, respectively (**Figure 3B**). In the GSE70770 cohort, the AUCs for risk scores at 1, 3, and 5 years were 0.671, 0.712, and 0.770, respectively (**Figure 3E**). The mRNA expression profiles of nine hub CCRGs were differently expressed in the different risk groups in MSKCC and GSE70770 (**Figures 3C, F**). In addition, the univariate and multivariate Cox regression analyses showed that the risk score was an independent prognostic predictor for RFS in TCGA-PRAD and MSKCC (**Table 2**).

**Table 1:** Univariate Cox regression analysis of differentially expressed CCRGs.

Gene name	HR	95% CI	*p*	Gene name	HR	95% CI	*p*
MYOCD	0.586	0.432–0.795	0.001	SCRIB	1.787	1.243–2.57	0.002
FGF10	0.554	0.335–0.916	0.021	MKI67	1.811	1.359–2.413	<0.001
FAM107A	0.672	0.538–0.839	<0.001	CDC20	1.943	1.551–2.434	<0.001
MEIS2	0.738	0.552–0.987	0.04	UBE2S	2.094	1.516–2.892	<0.001
ATP2B4	0.736	0.594–0.912	0.005	CCNL2	1.938	1.475–2.545	<0.001
EZH2	2.567	1.747–3.77	<0.001	KIFC1	1.993	1.524–2.606	<0.001
NR3C1	0.694	0.506–0.953	0.024	REC8	1.595	1.122–2.269	0.009
TACC1	0.714	0.548–0.931	0.013	RRM2	1.762	1.392–2.229	<0.001
KAT2A	2.13	1.524–2.976	<0.001	FOXM1	1.914	1.485–2.466	<0.001
EDN3	0.498	0.312–0.794	0.003	CDCA5	2.358	1.793–3.102	<0.001
TUBA4A	0.759	0.585–0.983	0.037	CCNB1	1.743	1.307–2.324	<0.001
PDCD2L	1.851	1.079–3.178	0.025	PRC1	2.373	1.682–3.347	<0.001
CHTF18	2.367	1.732–3.236	<0.001	CDK5RAP3	1.758	1.245–2.483	0.001
RUVBL1	2.097	1.371–3.206	0.001	CCNA2	1.917	1.46–2.517	<0.001
E2F5	1.783	1.169–2.72	0.007	SPC24	2.296	1.716–3.071	<0.001
TRIM35	0.586	0.354–0.968	0.037	KIF4A	2.318	1.716–3.132	<0.001
FLNA	0.818	0.695–0.963	0.016	MELK	1.975	1.458–2.677	<0.001
C11orf80	1.849	1.242–2.752	0.002	HMMR	1.92	1.406–2.623	<0.001
ZNF655	0.748	0.583–0.96	0.023	AURKB	1.955	1.505–2.54	<0.001
SAC3D1	1.635	1.177–2.271	0.003	ZWINT	1.562	1.175–2.077	0.002
ASNS	2.043	1.374–3.038	<0.001	MAPK12	1.736	1.267–2.378	0.001
BIRC5	1.828	1.453–2.299	<0.001	TOP2A	1.58	1.276–1.957	<0.001
EME2	1.885	1.45–2.451	<0.001	KIF20A	2.038	1.522–2.729	<0.001
CDCA8	2.253	1.598–3.178	<0.001	CDKN3	2.012	1.521–2.663	<0.001
MYBL2	1.713	1.388–2.113	<0.001	PTTG1	1.787	1.44–2.218	<0.001
CCNB2	1.898	1.42–2.539	<0.001	BTG2	0.804	0.653–0.99	0.040
UBE2C	1.639	1.362–1.972	<0.001	APP	0.789	0.628–0.989	0.040
HJURP	2.000	1.522–2.629	<0.001	NUSAP1	1.852	1.466–2.338	<0.001
TPX2	1.891	1.495–2.392	<0.001				

CCRGs, cell cycle-related genes.

**Table 2:** Univariate and multivariate Cox regression analysis in TCGA-PRAD and MSKCC cohorts.

Variables	Univariate Cox			Multivariate Cox		
	HR	95% CI	*p*-Value	HR	95% CI	*p*-Value
**TCGA**						
Age	1.031	0.999–1.063	0.055			
Gleason	2.232	1.798–2.771	<0.001	1.701	1.307–2.212	<0.001
T	2.536	1.69–3.804	<0.001	1.452	0.883–2.389	0.142
Risk score	2.717	2.053–3.596	<0.001	1.765	1.285–2.425	<0.001
**MSKCC**						
Age	1.018	0.97–1.069	0.460			
Gleason	3.708	2.576–5.339	<0.001	3.492	2.405–5.071	<0.001
T	1.527	0.861–2.708	0.148			
Risk score	8.079	2.008–32.498	0.003	3.749	1.048–13.414	0.042

TCGA-PRAD, The Cancer Genome Atlas-Prostate Cancer; MSKCC, Memorial Sloan Kettering Cancer Center.

### Differences in Genomic Alteration Profiles, Copy Number Variation, and Tumor Mutational Burden Between Cell Cycle-Related Gene Risk Groups

**Figure 4:**
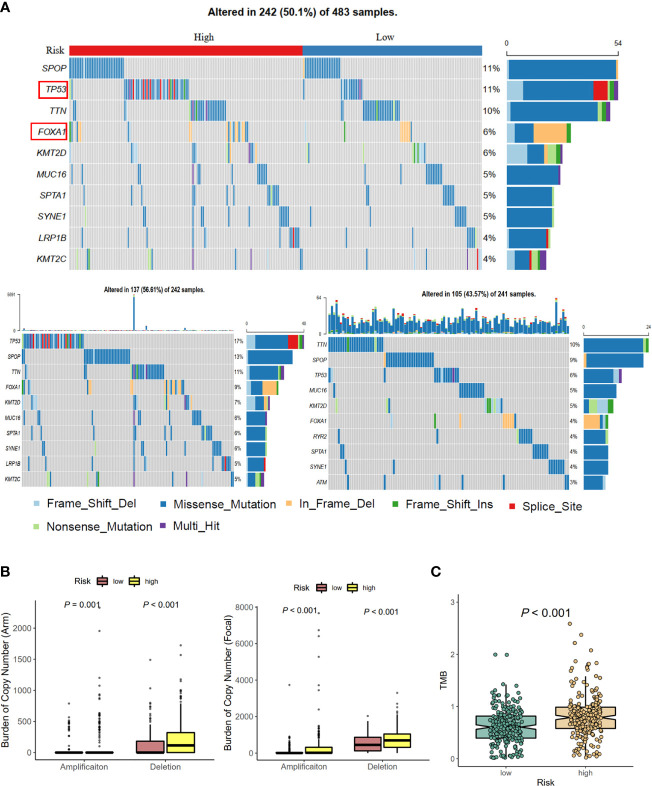
Mutational landscapes between high- and low-risk groups. **(A)** The distribution of frequently mutated genes in total, high-risk, and low-risk patients. **(B)** Arm-level, focal-level copy number amplification and deletion. **(C)** Tumor mutational burden difference.

Considering that genetic alterations were associated with the prognostic outcome of many cancers (13, 14), we compared the genomes of high- and low-risk groups. The top 10 genes with the highest mutation frequency in TCGA-PRAD cohort, high-risk group, and low-risk group are shown in **Figure 4A**. The mutation rates of TP53 and FOXA1 were 17% and 9% in the high-risk group, but 6% and 4% in the low-risk group, respectively, which indicated that CCRGs were probably associated with TP53 and FOXA1 pathways. For the copy number variation (CNV) status, the results demonstrated that the high-risk group had high burden of amplification (*p*_Arm-Amp_ = 0.001, *p*_Focal-Amp_ < 0.001) and deletion (*p*_Arm-del_ < 0.001, *p*_Focal-del_ < 0.001) at both the arm and focal levels (**Figure 4B**). Furthermore, tumor mutational burden (TMB) was significantly higher in the high-risk group than low-risk group (*p* < 0.001, **Figure 4C**).

### Functional Enrichment Between High-Risk and Low-Risk Groups

**Figure 5:**
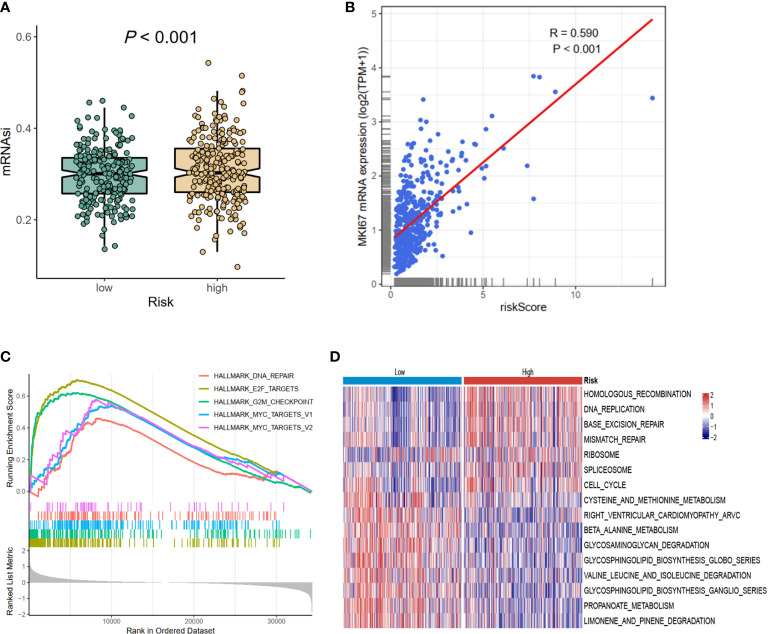
The differences of mRNAsi, MKI67, and potential biological pathways of high- and low-risk groups. **(A)** mRNAsi. **(B)** The correlations between MKI67 and risk score. **(C)** The hallmark enrichment based on the GSEA method. **(D)** The KEGG enrichment calculated by GSVA method. MSI, microsatellite instability; GSEA, Gene Set Enrichment Analysis; KEGG, Kyoto Encyclopedia of Genes and Genomes; GSVA, gene set variation analysis.

Considering the prognostic risk model was constructed based on CCRGs, which were associated with cell proliferation, then mRNAsi and MKI67 were analyzed. The results revealed reduced mRNAsi in the low-risk group (*p* < 0.001, **Figure 5A**), and the risk score was positively associated with MKI67 (R = 0.590, *p* < 0.001, **Figure 5B**). Next we investigated the potential functions in the high- and low-risk groups using GSEA and GSVA methods. Hallmark analysis showed that cell cycle-related pathways were enriched in the high-risk group (**Figure 5C**). Additionally, Kyoto Encyclopedia of Genes and Genomes (KEGG) analysis using GSVA showed that cell cycle-related pathways such as DNA_REPLICATION, MISMATCH_REPAIR, and CELL_CYCLE were also enriched in the high-risk group (**Figure 5D**). In summary, the results of mRNAsi, MKI67, and functional enrichment all demonstrated that the high-risk group was associated with cell cycle-related pathways, meaning that the prognostic signature could be represented by the CCRGs.

### Evaluation of Immune Cell Infiltration Characterization in Tumor Microenvironment

**Figure 6:**
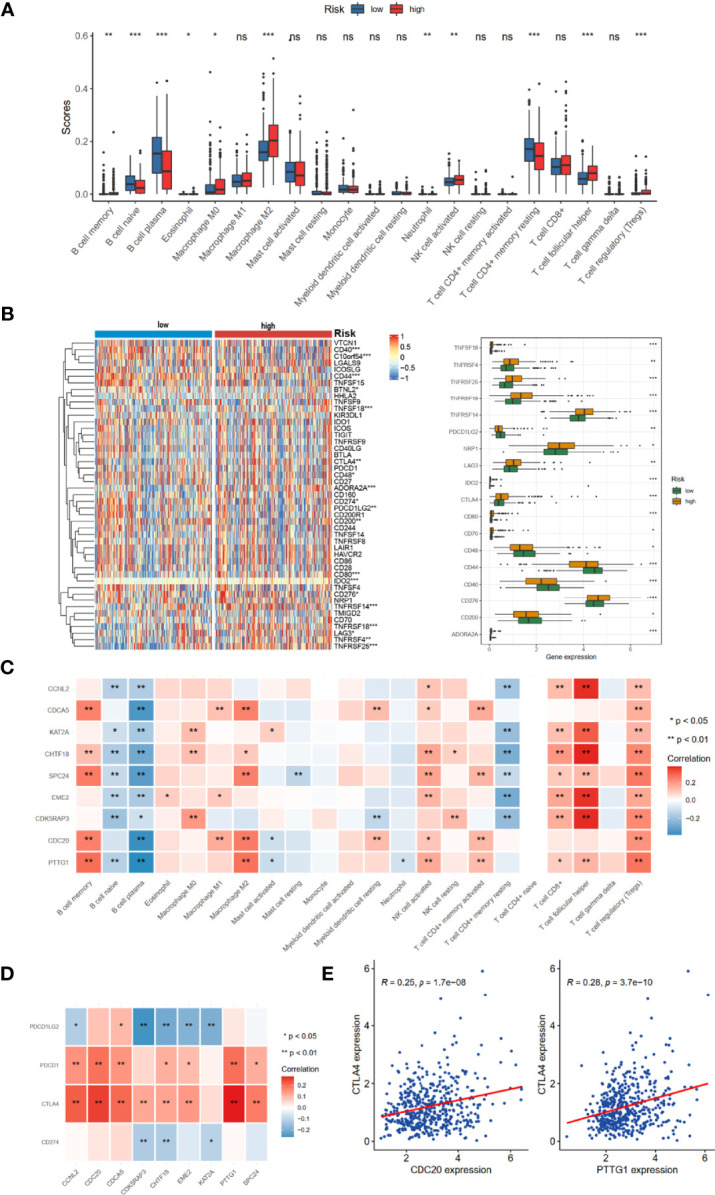
TME immune cell infiltration landscapes of different risk groups. **(A)** Differences of 24 TME infiltration cells based on CIBERSORT algorithm. **(B)** The mRNA expression profiles of common immune checkpoint genes. **(C)** The correlations between 9-risk genes and TME infiltration cell type. Red, positive; blue, negative. **(D)** The correlation between 9-risk genes and immune checkpoint molecules. **(E)** The correlation between REC, CTLA4, and PDCD1 (PD1). TME, tumor microenvironment. ns, no significance. *p < 0.05, **p < 0.01, ***p < 0.001.

The prognosis of tumor patients was significantly associated with the tumor microenvironment, especially with immune cells (15–17). Therefore, we hypothesized that the distribution of immune cells and expression of immune checkpoint genes would be significantly different in the two risk groups. We evaluated 24 immune cells by CIBERSORT method in normal and tumor samples. Low abundance immune cells were excluded; thus, only 21 immune cell types were assessed (**Figure 6A**). Of these, the high-risk group had a higher proportion of infiltration by B-cell memory cells, Macrophage M0, Macrophage M2, T-cell follicular helper, and T-cell regulatory (Tregs). The relationships between risk score and common immune checkpoint genes showed that many checkpoint genes were more highly expressed in the high-risk group, including PDL2 (PFCDL1G2), CD48, CD44, and CD200, while TNFRSF4, TNFRSF14, TNFRSF18, TNFRSF25, NRP1, LAG3, and CTLA4 were reduced (**Figure 6B**). Moreover, several members of the nine hub genes demonstrated significantly positive relationships with B-cell memory, Macrophage M2, T-cell follicular helper, and T-cell regulatory (Tregs) infiltration (**Figure 6C**).

We also found that the nine hub genes were positively associated with PDCD1 (PD-1) and CTLA4 **(** **Figure 6D**). Of these, CDC20 and PTTG1 were relatively significant (**Figure 6E**).

### Screening of Immunotherapeutic Benefits, Estimated IC50, and Candidate Drugs

**Figure 7:**
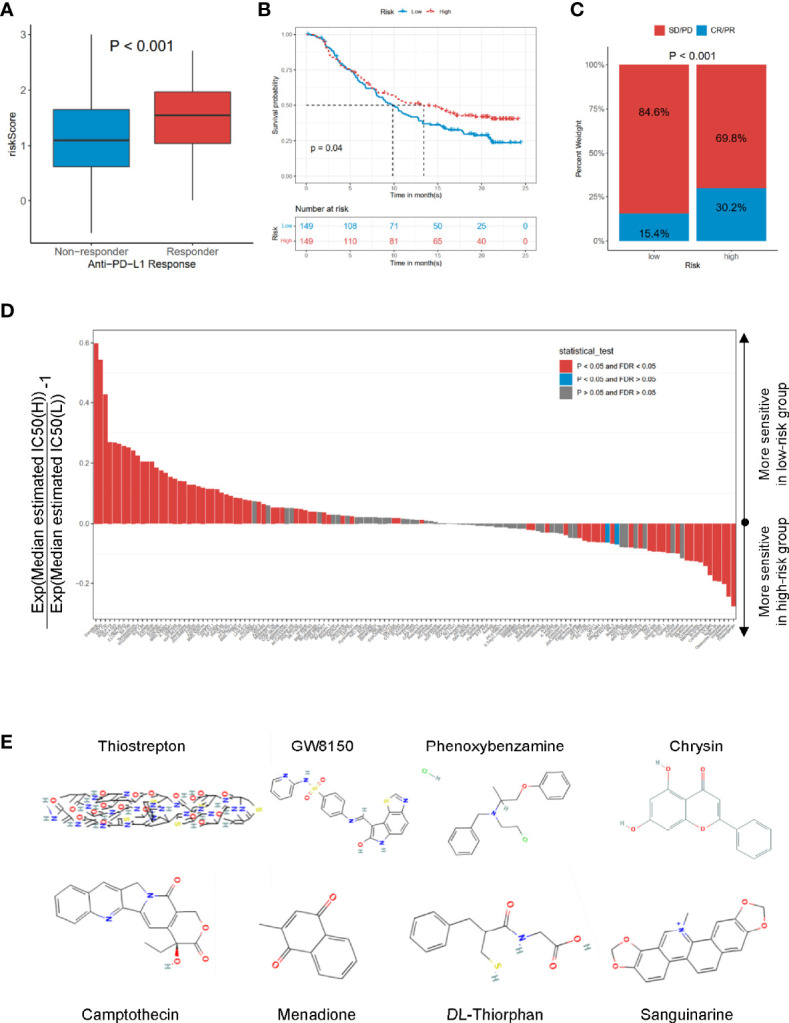
The roles of risk scores in the prediction of immuno-/chemotherapeutic benefits and candidate drugs. **(A)** Risk scores in high- and low-risk groups with different anti-PD-1 clinical response statuses. *p* < 0.001. **(B)** KM curves for high- and low-risk groups in the IMvigor210 cohort. Log-rank test, *p* = 0.032. **(C)** Rate of clinical response rate to anti-PDL1 immunotherapy in high- and low-risk groups in the IMvigor210 cohort. **(D)** Estimated IC50 for 138 compounds based on GDSC dataset. **(E)** 2D conformer of six significant candidate drugs. KM, Kaplan–Meier; GDSC, Genomics of Drug Sensitivity in Cancer.

To explore the potential performance of the risk signature on immunotherapeutic benefits, we analyzed the IMvigor210 cohort, who received anti-PD-L1 immunotherapy. Notably, a high response rate of anti-PD-L1 therapy was associated with a higher risk core (*p* < 0.001, **Figure 7A**). Moreover, the high-risk group had a more favorable survival rate (*p* = 0.04, **Figure 7B**) and objective response to anti-PD-L1 than the low-risk group (*p* < 0.001, **Figure 7C**). Based on the Genomics of Drug Sensitivity in Cancer (GDSC) dataset, the IC50 value of 138 compounds showed that the low-risk group might be more sensitive to 51 compounds, such as dasatinib, DMOG, and MG.132; and the patients in the high-risk group were likely sensitive to 30 drugs, such as thapsigargin, bleomycin, and vinblastine (false discovery rate (FDR) < 0.05, **Figure 7D**). In addition, the CMap dataset was utilized to predict the candidate drugs for the risk signature. The results showed that thiostrepton, GW8150, phenoxybenzamine, chrysin, camptothecin, menadione, dl-thiorphan, and sanguinarine were the most potential target drugs due to their enrichment scores (<−0.90) and *p* < 0.05 (**Table 3**), of which the 2D conformers are displayed in **Figure 7E**.

**Table 3:** Results of CMap analysis.

CMap name	Mean	n	Enrichment	*p*	Specificity
GW-8510	−0.770	4	−0.967	<0.001	0.0534
Phenoxybenzamine	−0.784	4	−0.949	<0.001	0.0091
Thiostrepton	−0.716	4	−0.902	<0.001	0.0093
Chrysin	−0.756	3	−0.957	<0.001	<0.001
Camptothecin	−0.767	3	−0.918	<0.001	0.1023
Menadione	−0.752	2	−0.967	0.002	0.0115
dl-Thiorphan	−0.724	2	−0.939	0.007	0.0171
Sanguinarine	−0.752	2	−0.932	0.009	0.0188

### The Characteristics of Hub Genes in the Oncomine and cBioPortal Datasets

**Figure 8:**
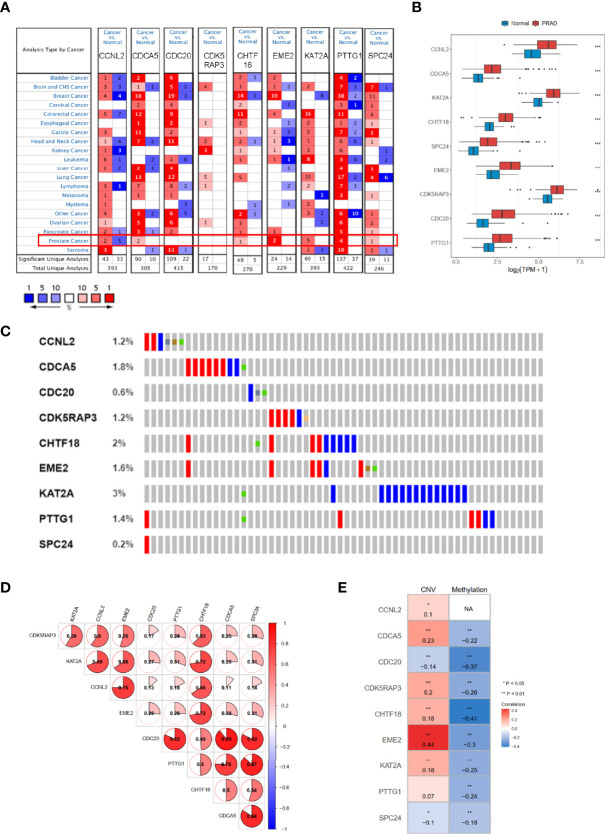
The mRNA expression patterns, genomic alterations, and methylation of nine hub genes. **(A)** The overview of nine hub genes in the Oncomine database. **(B)** The mRNA expression profiles of nine hub genes in TCGA-PRAD. **(C)** The genetic alterations of nine hub genes based on cBioPortal. **(D)** The correlations between these nine hub genes. **(E)** The correlations between mRNA expression and methylation, and copy number variation of nine hub genes. TCGA-PRAD, The Cancer Genome Atlas-Prostate Cancer. NA, not avaiable. *p < 0.05, **p < 0.01, ***p < 0.001.

We used Oncomine dataset to search the expression profiles of nine hub genes whose expression was increased (*p* < 0.05) in several types of cancer (**Figure 8A**), especially in PRAD patients, consistently with TCGA-PRAD cohort (**Figure 8B**). Moreover, the correlation among these hub genes was very high; for example, the correlation coefficients between PTTG1 and SPC24 was 0.87 (*p* < 0.05, **Figure 8D**). In addition, the genetic alteration status based on cBioPortal showed that the genetic alteration rates of these hub genes were less than 5% (**Figure 8C**), and several items belonged to the CNV change. The correlation between CNV and mRNA expression of EME2 was 0.44 (**Figure 8E**), while that of the others was less than 0.3. The methylation levels of nine genes (expect CCNL2, as data were absent from cBioPortal) were negatively associated with mRNA expression (*p* < 0.05, **Figure 8E**).

### Verification of Hub Genes Based on the Human Protein Atlas Datasetand RT-qPCR

**Figure 9:**
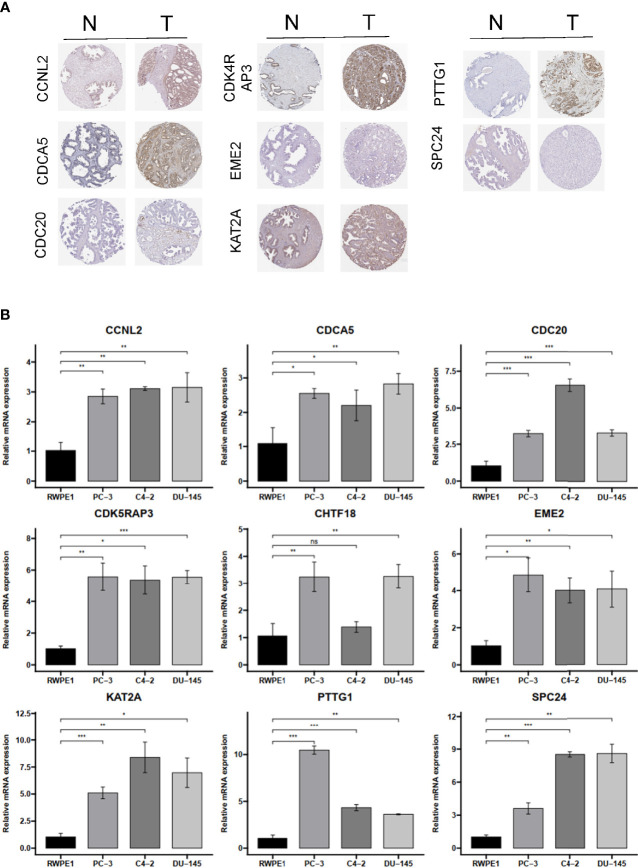
The IHC expression pattern based on HPA dataset and mRNA levels by qRT-PCR of CCNL2, CDCA5, CDC20, CDK4RAP3, EME2, KAT2A, PTTG1, REC8, and SPC24. **(A)** The IHC results of nine hub genes in prostate cancer and normal tissues based on HPA. **(B)** The mRNA level of nine hub genes in normal prostatic epithelial cell (RWPE-1) and prostate cancer cell lines. IHC, immunohistochemistry; HPA, Human Protein Atlas. ns, no significance. *p < 0.05, **p < 0.01, ***p < 0.001.

To verify the reliability of these hub genes, we detected the protein levels from the Human Protein Atlas (HPA) website in normal samples and PRAD tissues. The results showed that eight proteins (CCNL2, CDCA5, CDC20, CDK4RAP3, EME2, KAT2A, PTTG1, and SPC24; note that CHTF18 was absent) were significantly dysregulated in PRAD tissues compared with normal prostate tissues (**Figure 9A**). To further confirm the expression levels based on bioinformatics analysis, the mRNA expression profiles of these hub genes were detected by RT-qPCR from normal prostatic epithelial cells (RWPE-1) and prostate cancer cells (PC-3, C4-2, and DU-145). The results showed that all nine genes were significantly upregulated in PC-3 and DU-145 compared with RWPE-1 (**Figure 9B**), which were in accordance with the contents from TCGA and Oncomine datasets.

## Discussion

The cell cycle is an essentially biological process (18, 19). Under normal conditions, cells proliferate only in response to mitotic signals, which are more significant for normal organisms, while the proliferation of cancer cells is out of control (7). This suggested that the proliferation of cancer cells is associated with the dysregulation of proliferation-related signals, which could be controlled by the cell cycle. Several previous studies showed that dysregulated genes could give rise to the expression of key factors involved in cancer cell cycle. Therefore, we hypothesized that CCRGs have excellent performance in PRAD.

Recent studies have shown that high throughput RNA sequences and microarray profiles have been utilized to develop signatures for the outcome events of several clinical diseases (20). In our study, we firstly used the GSVA method to explore the differential hallmark pathways, and results showed that CCRGs were highly enriched in PRAD patients (**Figure 1A**). Based on the differential CCRGs, TCGA-PRAD patients could be classified into four clusters, which were significantly associated with RFS (**Figures 1C, D**). With regard to risk model construction, the random survival method was utilized to select the hub CCRGs based on variable importance (VIMP) and minimal depth (**Figures 2A, B**). Then nine hub genes were included in the next step, and multivariate Cox regression was applied to calculate risk scoring. The performances on the prediction of prognostic ability were validated in the MSKCC and GSE70770 datasets (**Figure 3**, **Table 2**). Moreover, hallmark and KEGG pathways (**Figures 5D, E**) showed that the high-risk group was enriched in cell cycle-related pathways. As genomic instability takes critical roles in the development of cancers (21, 22), then genetic alterations, such as mutation status, CNV load, and TMB, were analyzed in TCGA-PRAD cohort. The results showed that the high-risk group was significantly associated with high mutation rates, especially for TP53 and FOXA1 (**Figure 4A**), CNV load (**Figure 4B**), and TMB (**Figure 4C**), which may help tailor personalized treatment.

In recent years, numerous previous studies indicated that cell cycle gene signatures have the potential for evaluating immune cell infiltration, immune evasion, and immune responses (23–25). However, the relationship between cell cycle-related signatures and tumor immune situation in PRAD was not explored before. Therefore, we compared the immune cell abundance based on CIBERSORT and common checkpoint genes in TCGA-PRAD cohort. The high-risk group had inflammatory infiltrates with a higher proportion of M2 macrophages and T-cell regulatory (Tregs) (**Figure 6A**), which were associated with immune evasion (26, 27). Considering the essential roles of immune checkpoint genes in immunotherapy response, immune checkpoint genes were differently distributed between low- and high-risk groups, such as LAG3, CTLA4, NRP1, and CD276 (**Figure 6B**). Increased evidence demonstrated that single genes could reshape the tumor immune microenvironment and immune cell infiltration (28, 29). We revealed obviously positive correlations between these nine hub genes and the expression of B-cell memory cells, Macrophage M2, T-cell follicular helper, and T-cell regulatory (**Figure 6C**), consistent with the results of immune cell infiltration and risk groups. We also noted that most of these nine hub genes were positively correlated with PDCD1 and CTLA4 (**Figure 6D**), suggesting that these genes could mediate immune evasion and response to immunotherapy. In the IMvigor210 cohort with anti-PD-L1 immunotherapy, we found that patients with high-risk scores might benefit from anti-PD1 immunotherapy (**Figures 7A–C**).

Additionally, the analysis of drug sensitivity based on the GDSC dataset demonstrated that the CCRG risk signature might be useful for therapeutic applications. Several drugs, such as dasatinib, MG.132, lapatinib, and docetaxel, responded differently between the low- and high-risk groups (**Figure 7D**), which suggested that CCRGs influenced drug response to chemotherapy and targeted treatment. Fortunately, we found 8 drugs with *p* < 0.001 and enrichment less than −0.9 (**Table 3**), including GW-8510, phenoxybenzamine, thiostrepton, chrysin, camptothecin menadione, dl-thiorphan, and sanguinarine (**Figure 7E**, **Table 3**). GW-8510, an inhibitor of CDK2, has a similar effect to gemcitabine in inhibiting pancreatic cancer cells (30, 31). Phenoxybenzamine, an alpha blocker, has been used to inhibit histone deacetylases (32, 33) in human cancer cells. Thiostrepton, a natural antibiotic produced by bacteria, could induce upregulation of several heat shock proteins in various human cancer cells (34) and inhibit cancer stem cell growth (35). Chrysin could inhibit cancer growth *via* induction of apoptosis, alteration of the cell cycle, and inhibition of angiogenesis without causing any toxicity to normal cells (36, 37). Camptothecin (38), menadione (39, 40), dl-thiorphan (41), and sanguinarine (42) are also reported to have a strong relationship with cancer therapy. Moreover, many previous studies have been reported to have anticancer effects in prostate cancer cells, such as chrysin (43), phenoxybenzamine, thiostrepton (44), sanguinarine (45), camptothecin (46), menadione (47), and sanguinarine (48, 49). Finally, the mRNA expression profiles of nine hub genes were explored in TCGA-PRAD and validated in Oncomine, the HPA dataset, and prostate cancer cells based on RT-qPCR.

Although our study have comprehensive analyzed the CCRGs in PRAD, there were still some limitations. Firstly, we constructed and validated the CCRGs risk model only including public datasets, so more real-world data of PRAD are needed to verify the model’s prognostic performance. Secondly, we only validated the mRNA expression profiles of nine hub CCRGs in prostate cancer cells; more experimental studies are still necessary to confirm for clinical application, and more underlying mechanisms of these genes should be explored for further research.

In conclusion, this preliminary research of CCRGs in PRAD patients has profiled the expression levels and genetic alterations of CCRGs in PRAD, which may open up the development of novel drugs against PRAD. The prognostic model based on nine hub CCRGs was strongly correlated with high CNV load, TMB load, mRNAsi, infiltration of different types of immune cells, and chemo-/immunotherapy response, which may provide novel ideas for PRAD with patients chemo-/immunotherapy response.

## Materials and Methods

### Prostate Cancer Datasets and Samples

We used TCGA-PRAD cohort data, which includes mRNA expression data, clinicopathological features, and RFS data from the UCSC Xena (https://xenabrowser.net/datapages/) dataset. The raw read counts of RNA-seq were transformed into transcripts per kilobase million (TPM) values. The DNA methylation information and genetic mutations were collected from cBioPortal (50).

The external validation cohort, including the MSKCC (GSE21032) and GSE70770, were log2 normalized microarray matrix downloaded from http://cbio.mskcc.org/cancergenomics/prostate/data/ and Gene Expression Omnibus (GEO) (https://www.ncbi.nlm.nih.gov/geo/) dataset.

### Gene Set Variation Analysis, Gene Set Enrichment Analysis, Difference Analysis, and Consensus Clustering

The hallmark enrichment between PRAD and normal tissues and the KEGG enrichment for high and low risk were performed using the GSVA method. The hallmark enrichment between the high- and low-risk groups was generated by the GSEA method. Then, a panel of 1,875 CCRGs were recognized from MSigDB (51), as previously described (7). The differentially expressed CCRGs were calculated using the limma package in R with FDR < 0.05 and |logFC| > 0.5. Next, a consensus clustering algorithm was utilized to evaluate the prognostic ability of CCRGs using the ConsensusClusterPlus (52) R package.

### Calculation of the Cell Cycle-Related Gene Score for Prostate Cancer Patients

In TCGA-PRAD cohort, differentially expressed CCRGs were processed to univariate Cox regression analysis. The random forest algorithm was applied to select the candidate genes. Only genes included in the top 15 lists for both minimal depth and VIMP were selected. Then, PCA was also performed in R. Finally, the candidate genes were brought into the multivariate Cox regression analysis to build the prognosis model. The risk score of the CCRGs for each PRAD patient was computed based on the mRNA expression of selected CCRGs weighted by the multivariate Cox regression coefficient. Based on the median CCRG score, the patients were divided into high- and low-risk groups. Next, PCA was performed to explore the distribution of different risk groups. Finally, the prognostic values of CCRG score were evaluated using KM curve and the 1-, 3-, and 5-year ROC curves. A heatmap plot was utilized to display the expression profiles for the different groups.

As for the MSKCC and GSE70770 cohorts, the KM curve, ROC curve, and heatmap plot were also drawn to validate the prognostic performance.

### Identification of Somatic Alteration, Copy Number Variation, Tumor Mutational Burden, Tumor Stemness, and Immune Cell Infiltration in Different Groups

Somatic mutations data were downloaded from TCGA GDC (https://portal.gdc.cancer.gov/) using “TCGABiolinks” (53) R package. Copy number alterations data were calculated by GISTIC2.0 from GDAC Firehose (https://gdac.broadinstitute.org). The total number of mutations per megabyte of tumor tissue (TMB) was generated by the number of nonsynonymous mutations per million. Tumor stemness was previously study calculated by using the one-class logistic regression (OCLR) method (54). The CIBERSORT (55) method was processed to infer the proportion of immune cell infiltration in different tumor groups. The connectivity Map (CMap) (56) was utilized to screen potential candidate drugs.

### Screening of Immunotherapy, Chemotherapy Responses, and Potential Drugs

The IMvigor210 dataset was downloaded from IMvigor210CoreBiologies (57) to evaluate the predictive power of the CCRG scores. The estimated IC50 of 138 compounds in the GDSC (58) website was calculated using the “pRRophetic” (59) R package.

### The Characteristic of Nine Hub Cell Cycle-Related Genes

The mRNA expression levels of these nine hub CCRGs among pan-cancer were retrieved from Oncomine (https://www.oncomine.org/) with a threshold *p*-value <0.05 (60). The mRNA expression profiles of nine hub CCRGs in TCGA-PRAD were downloaded from UCSC Xena (61). The mutation alteration data, CNV, and DNA methylation data were downloaded from cBioPortal (http://www.cbioportal.org). The immunohistochemistry (IHC) results of nine hub genes were collected from the HPA (https://www.proteinatlas.org/) (62).

### Detection of the mRNA Levels of Hub Genes Using Real-Time Quantitative PCR

The human RWPE-1 cell lines were cultured in K-SFM (Biotecnómica, Porto, Portugal) medium, and human epithelial PRAD cell lines (PC-3, C4-2, and DU145) were cultured with DMEM 1640 medium. Total RNA, cDNA, and RT-qPCR were operated according to the manufacturer’s protocol. Independent experiments were performed in triplicate, and GAPDH served as the internal control. The primers are as follows:

CCNL2 gene 5′-GTACTCCGGGGTGCTCATC-3′ (sense) and 5′-GAGGTCGGTCTCTGTGTCG-3′ (antisense).

CDCA5 gene 5′-GAGGTCCCAGCGGAAATCAG-3′ (sense) and 5′-TCTTTAAGACGATGGGCTTTCTG-3′ (antisense).

KAT2A gene 5′-GCAAGGCCAATGAAACCTGTA-3′ (sense) and 5′-TCCAAGTGGGATACGTGGTCA-3′ (antisense).

SPC24 gene 5′-GCCTTCCGCGACATAGAGG-3′ (sense) and 5′-CCTGCTCCTTCGCATTGAGA-3′ (antisense).

EME2 gene 5′-CGCCGTTACCAAGGCTCTC-3′ (sense) and 5′-GCTGACCCGACTGAACTGC-3′ (antisense).

CHTF18 gene 5′-GAGCCGACTGACGGTCAAG-3′ (sense) and 5′-CGGTTGGTGAAGTCATCACTG-3′ (antisense).

CDK5RAP3 gene 5′-GAGTCTGGTGCTGACGATCC-3′ (sense) and 5′-TGTGAAGAGTATCGGCCAAAAAT-3′ (antisense).

CDC20 gene 5′-GCACAGTTCGCGTTCGAGA-3′ (sense) and 5′-CTGGATTTGCCAGGAGTTCGG-3′ (antisense).

PTTG1 gene 5′-ACCCGTGTGGTTGCTAAGG-3′ (sense) and 5′-ACGTGGTGTTGAAACTTGAGAT-3′ (antisense).

### Statistical Analysis

All statistical analyses were executed on the R platform (Version 4.1.0). A significant difference between the two groups was assessed using the Wilcoxon rank test, and among three or more groups, it was calculated using one-way ANOVA and Kruskal–Wallis tests. In addition, KM curves were produced, and a log-rank test was used, together with hazard ratios (HR) with 95% CI using univariate and multivariate Cox regression analyses, as well as correlation tests with Spearman’s method. All statistical *p*-values were two-sided, with *p* < 0.05 statistically significant.

## Data Availability Statement

The data about TCGA, GEO, CMap and HPA dataset are publicly available, other original contributions presented in the study are included in the article. Further inquiries can be directed to the corresponding author.

## Author Contributions

ZL and RP: conceptualization, supervision, methodology, and writing—review and editing. WL and YL: data curation, formal analysis, and writing—original draft. ZL, RP, and YL: validation, visualization, and investigation. All authors contributed to the article and approved the submitted version.

## Conflict of Interest

The authors declare that the research was conducted in the absence of any commercial or financial relationships that could be construed as a potential conflict of interest.

## Publisher’s Note

All claims expressed in this article are solely those of the authors and do not necessarily represent those of their affiliated organizations, or those of the publisher, the editors and the reviewers. Any product that may be evaluated in this article, or claim that may be made by its manufacturer, is not guaranteed or endorsed by the publisher.
